# The Prediction of High-Temperature Bulging Deformations in Non-Uniform Welded Tubes and Its Application to Complex-Shaped Tubular Parts

**DOI:** 10.3390/ma18122882

**Published:** 2025-06-18

**Authors:** Zhenyu Zhang, Yanli Lin, Xianggang Ruan, Jiangkai Liang, Tianyu Wang, Junzhuo Wang, Zhubin He

**Affiliations:** State Key Laboratory of High-Performance Precision Manufacturing, School of Mechanical Engineering, Dalian University of Technology, Dalian 116024, China; zzyabc321zzy@163.com (Z.Z.); ruanxg530@163.com (X.R.); 15535368969@163.com (J.L.); 467159478@mail.dlut.edu.cn (T.W.); luckywjz2025@163.com (J.W.); hezb@dlut.edu.cn (Z.H.)

**Keywords:** non-uniform welded tube, Johnson–Cook model, modified Johnson–Cook model, finite element analysis, high-temperature deformation behavior

## Abstract

Boron steel welded tubes show strong potential as blanks in the integrated hot gas forming–quenching process for fabricating complex thin-walled automotive parts. Nonetheless, the non-uniform characteristics of the base metal and the weld in the high-heat welded tube can result in uneven deformation during the bulging process. This inconsistency hampers precise predictions of the deformation behavior of the welded tubes at high temperatures. Accordingly, this research explored the flow characteristics and mechanical properties of PHS1500 boron steel welded tubes. This research was conducted at 850 °C and 900 °C, with strain rates of 0.01 s^−1^–1 s^−1^. The Johnson–Cook model was modified for both the base metal and the weld using experimental stress–strain data. Meanwhile, to assess the model precisions, the correlation coefficient *r* and the average absolute relative error (*AARE*) were employed. Finally, hot gas forming of PHS1500 boron steel welded tubular parts with complex shapes was predicted through a finite element analysis. This research showed a positive correlation of the strain rate with both the yield and tensile strengths in the base metal and the weld. The average yield strength and tensile strength of the weld were 12.8% and 3.9% higher than those of the base metal, respectively. The *r* and *AARE* of the modified Johnson–Cook constitutive model for the base metal’s and the weld’s flow stress were 0.99 and 2.23% and 0.982 and 5.31%, respectively. The maximum deviation in the predictions of the distribution of the wall thickness of a typical cross-section of the formed complex-shaped tubular parts was less than 8%.

## 1. Introduction

Replacing traditional steel with high-strength steel in body structures is essential for lighter, safer vehicle designs [1,2,3]. Boron steel sheets, represented by 22MnB5, are widely used in lightweight body structures [4,5]. During hot stamping of boron steel, the sheet is preheated to 950 °C for a minimum of 5 min to achieve complete austenization. It is then rapidly moved to the forming die, where stamping and in-die quenching are performed. The formed boron steel parts form a complete martensite structure, and its tensile strength can exceed 1400 MPa [6]. Swift advancements in the automotive industry and growing emphasis on energy conservation and emission reductions have led to heightened demands for component integration [7,8]. Thin-walled welded tubes offer benefits such as cost-effective production, high efficiency, and consistent quality [9,10]. Boron steel welded tubes, processed via integrated hot gas forming–quenching, show substantial potential for fabricating complex, thin-walled automotive parts. At elevated temperatures, the non-uniform characteristics of the base metal and the weld in a welded tube cause uneven deformations during bulging, thereby affecting precise predictions of a tube’s bulging deformation behavior.

A finite element analysis (FEA) can effectively predict material deformation, aiding metal forming processes [11,12,13]. In a finite element analysis, the constitutive model plays a critical role in capturing the flow behaviors of the materials. The precision of the constitutive model substantially impacts the dependability of the simulation outcomes. Researchers have created various models to describe the thermoplastic rheological behavior of metals, including phenomenological, physical quantity, and machine learning models. Phenomenological models are simpler to develop and less parameter-dependent than physical quantity or machine learning models. Therefore, this kind of constitutive model has been more widely promoted and applied. The Johnson–Cook (JC) formulation, a phenomenological constitutive model, sees widespread applications, particularly for modeling metals.

The Johnson–Cook constitutive model characterizes the flow stress as a multiplicative combination of factors, commonly used to study the deformation of metals at elevated temperatures. While the conventional Johnson–Cook model provides a useful framework, it falls short by treating strain hardening, the strain rate sensitivity, and thermal softening as isolated variables. This oversimplification leads to noticeable gaps between the theoretical predictions and actual experimental measurements of the flow stress. Lin et al. [14] refined the conventional Johnson–Cook model to address this issue while investigating the hot deformation characteristics of alloy steel. This model integrated the interdependent relationship between the strain, the strain rate, and the temperature. This research ultimately demonstrated that this optimized JC model provides precise predictions of the flow stress behavior in alloy steel under high-temperature conditions. In addition, the traditional Johnson–Cook model fails to accurately characterize the non-linear positive correlation between the flow stress and the strain rate [15]. Recognizing this limitation, Shin et al. [16] presented an adaptation of the Johnson–Cook constitutive model. Their refined formulation successfully captures both the linear and exponential rise in the flow stress relative to the logarithmic strain rate while precisely modeling the thermal softening effects in metals under extreme heat. This advancement proves particularly valuable when analyzing the high-temperature deformation characteristics of copper and tantalum, where the modified JC model demonstrates far greater predictive reliability than its conventional counterpart. Researchers have extended the modified JC framework to investigate the high-temperature rheological properties of various metals [17,18,19].

By adapting the correction methods for the JC constitutive model used to other metals, scholars have characterized the high-temperature rheological behavior of boron steel. Ji et al. [20] investigated the thermal rheological properties of 22MnB5 boron steel. They incorporated a temperature sensitivity factor, proposing a modified Johnson–Cook model. To assess the precision of the model, they utilized the correlation coefficient (*r*) as an evaluation metric. The results demonstrated an impressive *r*-value of 0.985, providing compelling evidence that the enhanced JC model delivers a superior predictive performance compared to that of its traditional counterpart. Li et al. [21] improved the Johnson–Cook model, factoring in the coupled effects of hardening, rate sensitivity, and thermal weakening on the yield strength. Their research indicates that the corrected model significantly improves predictions of the flow behavior of B1500HS boron steels at elevated temperatures. At elevated temperatures, boron steel welded tubes exhibit performance variations between the base metal and the weld. Hence, a comprehensive comparison of the flow behavior and mechanical attributes in the base metal versus those in the weld zone under thermo-mechanical processing conditions is crucial. However, very rarely has a high-temperature constitutive model of boron steel welded tubes been established and modified according to the characteristics of heterogeneous materials.

This study examines PHS1500 boron steel welded tubes as its primary focus. Through hardness testing, the width of the weld zone is identified, while micro-sampling techniques are employed to extract tensile specimens from the weld region. This study analyzes the tensile deformation of the base metal and the weld at 850–900 °C and strain rates of 0.01 s^−1^–1 s^−1^. Through the experimental findings, a Johnson–Cook model for the base metal and the weld was developed and modified. Concurrently, the model precision was assessed using two quantitative metrics. A finite element analysis was employed to forecast the hot gas bulging in a PHS1500 boron steel welded tube. This research offers theoretical insights for the precise formation of complex integral components in boron steel welded tubes.

## 2. Experiments

### 2.1. The Materials

This study utilized a PHS1500 boron steel sheet from the Ben Gang Group (Benxi, China), with its detailed chemical composition in Table 1. Following rolling, the sheet undergoes high-frequency electric resistance welding (HF-ERW) to form a tube [22]. The welding time of a PHS1500 tube is 300 ms, the welding current range is 7 kA~8.8 kA, and the current frequency range is 350~400 kHz. This tube features an outer diameter measuring 63.5 mm and a wall thickness of 1.4 mm.

The flow characteristics of the base metal and the weld can significantly differ under high-temperature conditions. This difference stems from the unique microstructure and grain dimensions of both materials at room temperature. The base metal area has a relatively uniform, tied structure, and the weld zone may form coarse grains or complex phase change structures due to rapid heating and cooling during the welding process, resulting in its strength, toughness, and creep resistance under high-temperature conditions significantly differing from those of the base metal [23]. Additionally, the inconsistent allocation of residual stress and strain within the weld area may amplify the difference between the base metal and the weld [24]. Studying the flow behavior of the base metal and the weld at high temperatures aids in the effective application of HF-ERW welded tubes in the hot gas bulging process.

**Figure 1:**
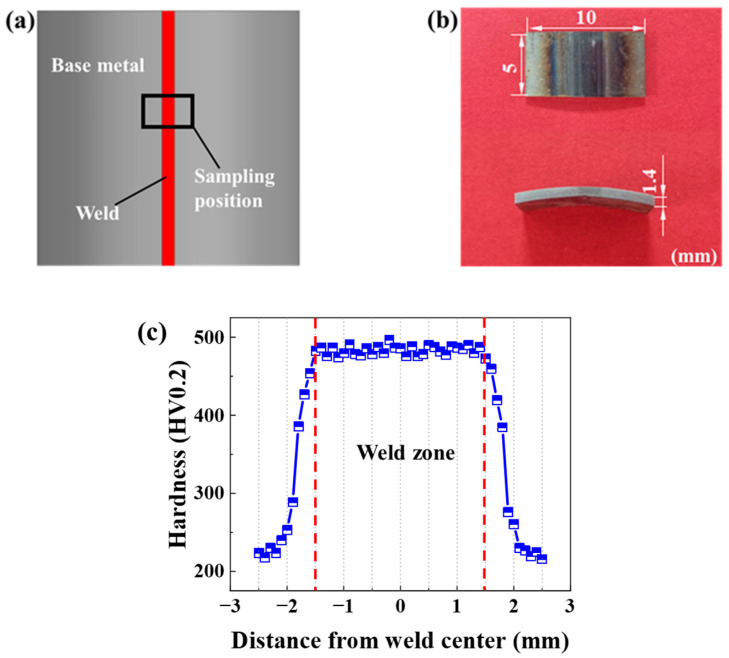
The sampling location of the hardness specimen and the specimen’s Vickers hardness distribution: (**a**) the hardness specimen’s sampling location; (**b**) size; (**c**) and Vickers hardness distribution.

The width of the weld zone was determined by cutting a hardness sample from the welded tube using the wire cutting method, as illustrated in Figure 1. The hardness sample underwent hot inlay treatment for subsequent grinding and polishing. The sample’s surface was initially deburred using coarse sandpaper, followed by sequential grinding with 400#, 800#, 1200#, and 1600# metallographic sandpaper. After grinding the sample’s surface to remove visible scratches, mechanical polishing was performed using an YMPZ-1–250 automatic metallographic grinding machine (Shenyang (Liaoning) Kejing Automation Equipment Co., Ltd., Shenyang, China). The hardness of the sample was evaluated using an HVS-1000 microhardness tester (Laizhou (Shandong) Metalreader Testing Instrument Co., Ltd., Laizhou, China) after polishing. Hardness measurements were taken at 0.1 mm intervals perpendicular to the weld center, determining the weld zone’s width to be approximately 3 mm.

### 2.2. Hot Uniaxial Tensile Tests

**Figure 2:**
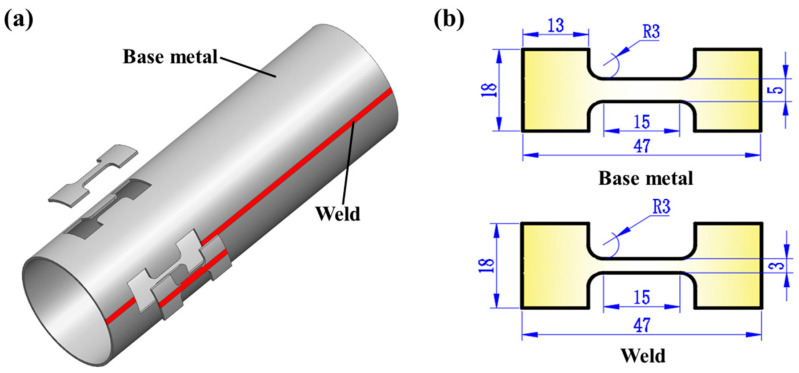
The sampling location and specimen size of the tensile specimen: (**a**) the specimen’s sampling location; (**b**) the specimen’s size.

Based on the width of the weld zone determined from the hardness test, samples are taken from both the base metal and the weld in the axial direction of the welded tube. According to the ASTM-E21 standard [25], tensile specimens are prepared using the wire cutting method, as illustrated in Figure 2.

**Figure 3:**
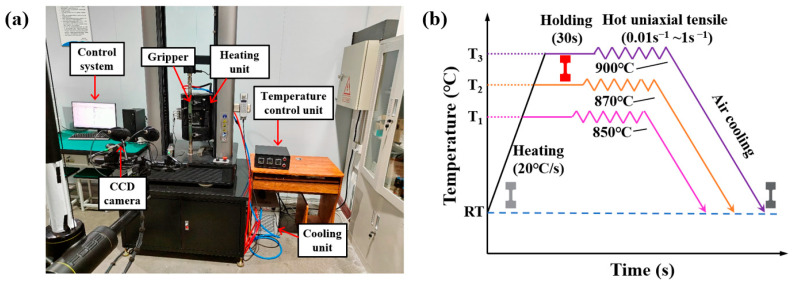
High-temperature tensile test: (**a**) experimental setup; (**b**) test program.

To eliminate wire cutting traces in the parallel section of the sample and avoid the influence of stress concentration on the tensile properties, sandpaper was used to polish the parallel section and the fillet area of the sample. The high-temperature tensile test setup for the welded tube’s base metal and weld comprises a heating unit, a temperature control unit, a water cooling unit, a strain acquisition unit, and a control system, as illustrated in Figure 3a. The sample is heated and monitored using a heating furnace and the temperature control unit, and the high-temperature fixture of the tensile testing machine is cooled by the water cooling unit. The CCD camera, key to digital image correlation (DIC), captures field data on the strain as the sample undergoes tensile deformation. The tensile testing machine records the load during the tensile process for the sample, while the control system simultaneously calculates the stress of the sample and the strain in real time.

Boron steel can be completely austenitized at 850 °C [26]. In this experiment, the material flow was assessed at three forming temperatures: 850 °C, 870 °C, and 900 °C. Figure 3b illustrates the high-temperature tensile test setup for the samples. During the tensile test at elevated temperatures, the specimen is first heated to the target level at 20 °C/s. It is maintained for 30 s to remove thermal gradients. Subsequently, the uniaxial tensile test is conducted using the LE5105 material tension tester (Lishi (Shanghai) Scientific Instrument Co., Ltd., Shanghai, China) at strain rates of 0.01 s^−1^, 0.1 s^−1^, and 1 s^−1^. Once fractured, the sample is removed from the heating furnace for air cooling.

## 3. Results and Discussion

### 3.1. The Flow Behavior of the Base Metal and the Weld at Elevated Temperature

**Figure 4:**
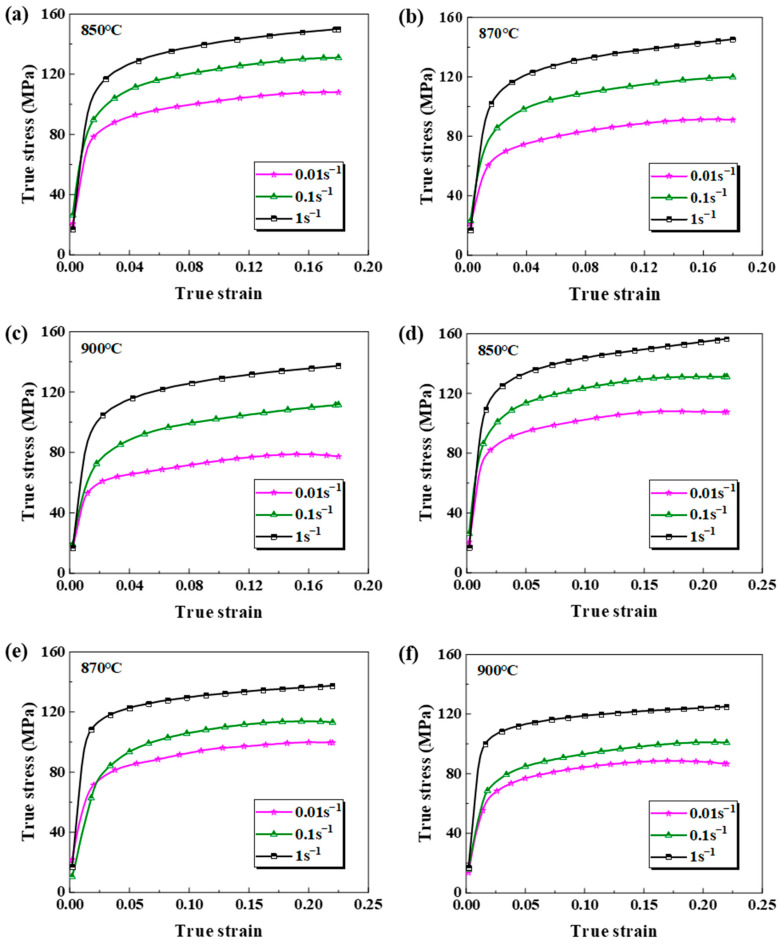
The true stress–strain curves for the base metal and the weld: (**a**) the base metal at 850 °C; (**b**) the base metal at 870 °C; (**c**) the base metal at 900 °C; (**d**) the weld at 850 °C; (**e**) the weld at 870 °C; (**f**) the weld at 900 °C.

As is illustrated in Figure 4, the flow curve of the base metal and the weld exhibits several deformation phases. In the elastic deformation stage, the curve shows a linear stress–strain relationship, which conforms to Hooke’s law. When the material yields and undergoes plastic deformation, the stress–strain relationship becomes non-linear. During the initial phase of plastic deformation, significant work hardening occurs, evidenced by a gradual increase in the flow stress with increasing strain. Work hardening is mainly caused by the accumulation of dislocation and lattice distortion. These mechanisms lead to further deformation of the material, requiring higher external forces [27]. During the advanced phase of plastic deformation, the dynamic recrystallization-induced softening and the continuous deformation-induced hardening of the matrix reach equilibrium, stabilizing the flow stress curve until necking fracture occurs.

Under a consistent strain rate, elevated temperatures reduce the yield strength in both the substrate material and the weld. The internal mechanism of this phenomenon is that the intensification of atomic thermal motion first weakens the lattice’s binding force, and weakening of the lattice’s binding force promotes dynamic recovery and the dynamic recrystallization process, which ultimately effectively offsets the cumulative effect of the dislocation density and reduces the deformation resistance of the material [28].

At a fixed temperature, the base metal and the weld both show strain rate hardening. For instance, at 850 °C and a 0.12 strain, the flow stress elevates from 101 MPa to 144 MPa when the strain rate accelerates from 0.01 s^−1^ to 1 s^−1^. This represents a 25.7% increase to 127 MPa at 0.1 s^−1^ and a further 13.4% increase to 144 MPa at 1 s^−1^. The weld showed a similar trend; that is, the flow stress increased from 108 MPa (0.01 s^−1^) to 134 MPa (0.1 s^−1^), an increase of 24.1%, and reached 148 MPa at 1 s^−1^, which was 10.4% higher than that at 0.1 s^−1^. At a 0.12 strain, both in the base material and the weld, the strain rate sensitivity initially increases before decreasing across the 0.01 s^−1^ to 1 s^−1^ strain rate spectrum.

**Figure 5:**
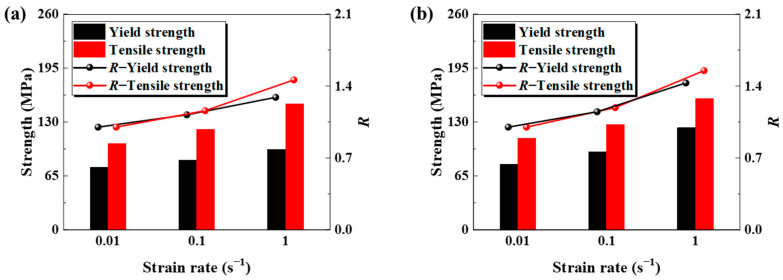
Changes in yield strength, tensile strength, and dynamic factor *R* of base metal and weld at 850 °C: (**a**) base metal; (**b**) weld.

To gain deeper insights into how the base metal and weld materials deform under varying strain rates, the dynamic factor *R* is employed as a measure of the strain rate sensitivity. The expression for the correlation is as indicated below:

(1) $$R=\frac{\sigma_{0}+\Delta \sigma }{\sigma_{0}}$$

where *σ*_0_ is the strength of the material at the selected reference strain rate, and Δ*σ* is the amount of change in the strength of the material at the different selected strain rates. Quantitative profiling of the elevated-temperature mechanical attributes in both the base metals and welds can be facilitated by a dynamic factor analysis. At 850 °C, both the base metal and the weld exhibit positive strain rate sensitivity, with their yield and tensile strengths increasing as the strain rate rises, as illustrated in Figure 5. The yield strength of the base metal rose from 76 MPa at 0.01 s^−1^ to 97 MPa at 1 s^−1^, whereas the yield strength of the weld increased more substantially from 79 MPa to 123 MPa under identical conditions. The tensile strength showed a similar trend: the base metal increased from 104 MPa (0.01 s^−1^) to 152 MPa (1 s^−1^), while the weld increased from 110 MPa to 158 MPa. Statistics indicate that the weld consistently exhibits a superior strength across all test conditions, with the average yield and tensile strengths surpassing the base metal by 12.8% and 3.9%, respectively.

The variation law for the dynamic factor *R* of the base metal and the weld at 850 °C was obtained, and the increase in *R* was more significant at a higher strain rate. The *R* value for the yield strength of the base metal rose from 1.12 at a strain rate of 0.1 s^−1^ to 1.29 at 1 s^−1^, while the *R* value for the tensile strength increased from 1.23 to 1.42. The *R* value of the weld follows the same pattern, with the yield strength rising from 1.15 to 1.43 and the tensile strength increasing from 1.26 to 1.57. The comparative analysis indicates that the tensile strength of both the base metal and the weld exhibits approximately 11% greater sensitivity to the strain rate compared to the yield strength across various experimental conditions.

The strain rate sensitivity coefficient *M* quantifies the flow stress response to variations in the strain rate, defined as

(2) $$M=\frac{\partial \ln\sigma }{\partial \ln\dot{\varepsilon }}$$

where *σ* is the flow stress, and $\dot{\varepsilon }$ is the strain rate.

**Figure 6:**
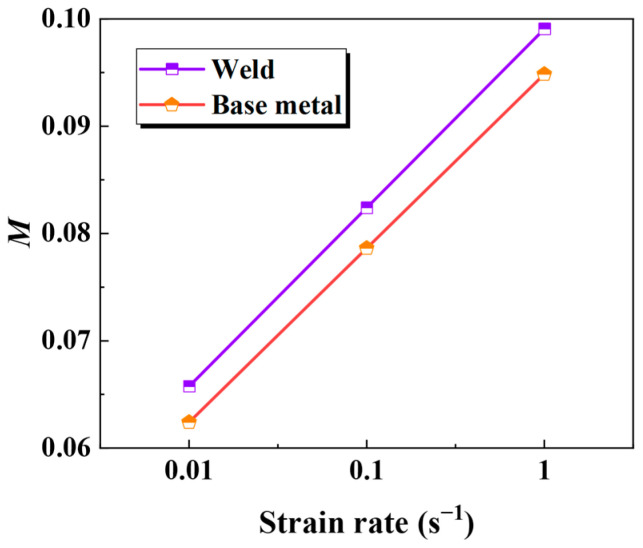
Sensitivity factor of strain rate between base metal and weld at 850 °C.

Figure 6 displays the strain rate sensitivities of the base metal and the weld at 850 °C, which is based on the data in Figure 4. This sensitivity coefficient rises linearly with the strain rate for the base metal and the weld.

### 3.2. The Johnson–Cook Constitutive Model

The analysis from the prior section reveals distinct mechanical characteristics for the base metal and the weld of the PHS1500 tube under elevated temperatures. Accordingly, characterizing the plastic deformation of the welded tube necessitates separate constitutive models for the base metal and the weld. The simple form and adaptability of the JC model make it a popular choice for modeling materials’ behavior under dynamic loads [29].

The JC constitutive framework is employed to depict the true stress–true strain curves for the metal across various strain rates and temperature conditions. The equation is as follows:

(3) $$\sigma =(A+B\varepsilon^{n})(1+C\ln(\dot{\varepsilon }/{\dot{\varepsilon }}_{0}))(1-{\left((T-T_{r})/(T_{m}-T_{r})\right)}^{m})$$

where *σ* is the stress; ε is the strain; $\dot{\varepsilon }$_0_ denotes the standard strain rate; and the parameters *A*, *B*, *C*, *n*, and *m* serve as material-specific constants, each carrying distinct physical implications. Here, *T* denotes the current temperature, while *Tr* stands for the chosen reference temperature. Additionally, *T_m_* indicates the material’s melting point.

The right-hand side of the constitutive equation comprises three primary components: a strain-enhanced term, with the parameters derived from the stress–strain curves from the thermal tensile experiment; a strain-rate-sensitive term, determined from the stress–strain plots at varying strain rates; and a temperature-softening term.

The parameters for the JC constitutive model of the base metal were derived through formula transformation and linear fitting, using a strain rate of 0.01 s^−1^ and a temperature of 1123 K as the deformation conditions, as detailed in Table 2.

The quantitative method assesses the model’s prediction accuracy more objectively. The predictive performance of the model is assessed using two metrics: the correlation coefficient *r* and the *AARE*. This method offers a more comprehensive evaluation of how well the predictions match the experimental data than a single error measure. The two parameters are expressed as

(4) $$r=\frac{\sum_{i=1}^{N}\left(\sigma_{e}-{\bar{\sigma }}_{e}\right)\left(\sigma_{p}-{\bar{\sigma }}_{p}\right)}{\sqrt{\sum_{i=1}^{N}{\left(\sigma_{e}-{\bar{\sigma }}_{e}\right)}^{2}\sum_{i=1}^{N}{\left(\sigma_{p}-{\bar{\sigma }}_{p}\right)}^{2}}}$$

(5) $$AARE=\frac{1}{N}\sum_{i=1}^{N}\left|\frac{\sigma_{e}-\sigma_{p}}{\sigma_{e}}\right|\times 100\%$$

where *σ_e_* and *σ_p_* are the experimental and predicted values of the true stress, respectively; $\bar{\sigma }$*_e_* and $\bar{\sigma }$*_p_* are the mean values of *σ_e_* and *σ_p_*, respectively; *i* is the sample number; and *N* is the total number of samples.

**Figure 7:**
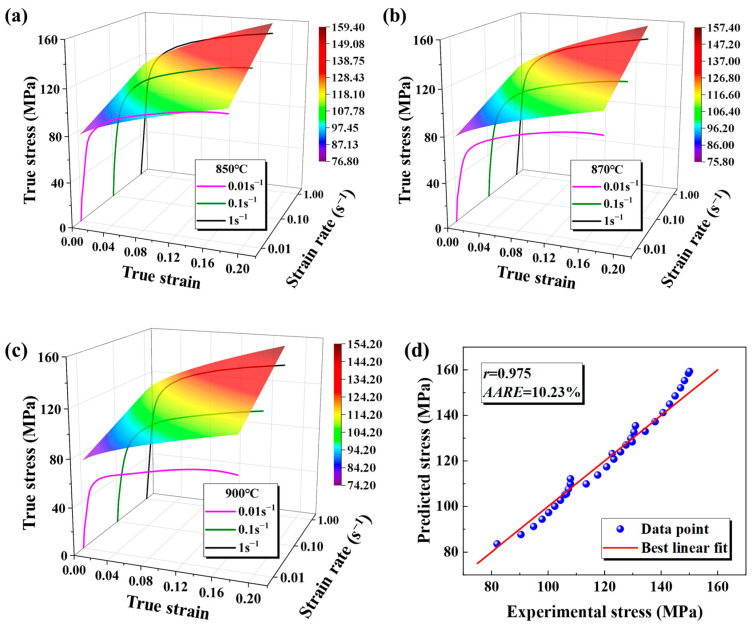
The fitting results of the JC constitutive model under different conditions of the base metal and the prediction accuracy of the model: (**a**) 850 °C; (**b**) 870 °C; (**c**) 900 °C; (**d**) the prediction accuracy of the JC constitutive model.

**Figure 8:**
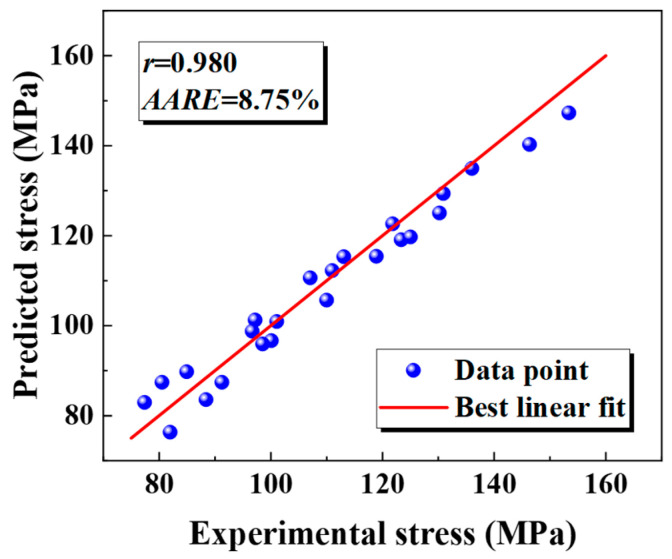
The prediction accuracy of the JC constitutive model for the weld.

Figure 7 contrasts the experimental stress–strain data for the base metal at 850 °C (0.01 s^−1^–1 s^−1^) with the JC model’s predictions. The solid line shows the experimental flow stress across strains, while the curved surface displays the predicted values. The fitted JC constitutive model significantly deviates from the experimental curve, with the deviations increasing at higher temperatures. The deviation in the base metal at different temperatures was quantitatively analyzed. The base metal had an *r*-value of 0.975 and a 10.23% *AARE*, while the weld showed a 0.980 *r*-value and an 8.75% *AARE*, as depicted in Figure 8. The JC constitutive model fits the weld behavior poorly.

This deviation primarily arises from the fixed nature of the model parameters and the variable characteristics of the dynamic factor *R* and the strain rate sensitivity coefficient *M* [30]. Although theoretically constant, the strain rate sensitivity coefficient exhibits a significant linear relationship with the strain rate in the experimental observations. Thus, the Johnson–Cook model incompletely captures the rheology of materials exhibiting strain rate sensitivity.

### 3.3. The Development of the Modified Johnson–Cook Constitutive Model

The Johnson–Cook constitutive model, a classic in its field, is commonly used to describe how materials respond to strain hardening, the strain rate, and thermal softening. However, its core assumption considers the temperature and the strain rate as separate variables, which remains a limitation. This assumption fails to accurately represent the interaction between the temperature and the strain rate in materials with significant coupling effects, thereby limiting its applicability in complex conditions [31].

The modified JC model addresses this issue by integrating the influences of temperature and strain rate within the flow stress calculations through coupling terms [14]. This model incorporates the temperature–strain rate interplay within the flow stress estimations via mathematical formulations, thereby mitigating the shortcomings of the earlier model:

(6) $$\sigma =(A_{1}+B_{1}\varepsilon +B_{2}\varepsilon^{2})(1+C_{1}\ln\dot{\varepsilon }*)\exp[(\lambda_{1}+\lambda_{2}\ln\dot{\varepsilon }*)(T-T_{r})$$

where *A*_1_, *B*_1_, *B*_2_, *C*_1_, *λ*_1_, *λ*_2_ are constants, and the others are the same as in the JC model.

Strain rates of 0.01 s^−1^ and 1123 K were likewise chosen as the benchmark deformation parameters for the model solution. The modified JC model is given by

(7) $$\sigma =(A_{1}+B_{1}\varepsilon +B_{2}\varepsilon^{2})$$

**Figure 9:**
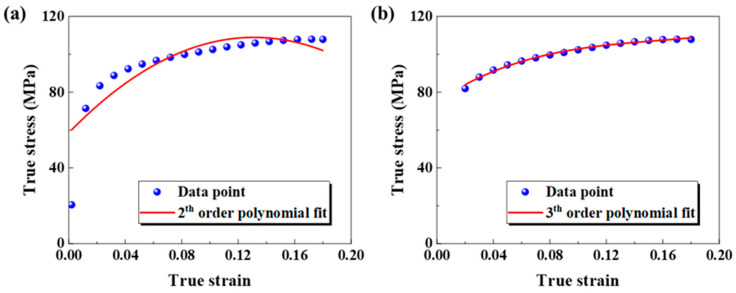
Fitting of stress–strain curves under reference conditions: (**a**) quadratic function fitting; (**b**) cubic function fitting.

At a reference strain rate and a reference temperature, the flow curves of the base metal were plotted as a scatter plot and then fitted using a quadratic polynomial equation, and the quadratic polynomial in the formula was used for fitting. Figure 9a illustrates the outcomes, indicating an imperfect fit. To enhance the precision of the model, this study elevates the polynomial order of the initial term, as illustrated in Figure 9b. Additionally, only data points after the yield point on the curve are incorporated, as expressed by the following equation:

(8) $$\sigma =(A_{1}+B_{1}\varepsilon +B_{2}\varepsilon^{2}+B_{3}\varepsilon^{3})$$

Based on the polynomial fitting accuracy, the modified JC model is defined as

(9) $$\sigma =(A_{1}+B_{1}\varepsilon +B_{2}\varepsilon^{2}+B_{3}\varepsilon^{3})(1+C_{1}\ln\dot{\varepsilon }*)\exp[(\lambda_{1}+\lambda_{2}\ln\dot{\varepsilon }*)(T-T_{r})]$$

According to the curve fitting effect in Figure 9b, *A*_1_ = 75.06529, *B*_1_ = 500.3209, *B*_2_ = −2831.127, and *B*_3_ = 6062.14381 were obtained.

Under the condition of the reference temperature, the formula can be transformed into

(10) $$\frac{\sigma }{\left(A_{1}+B_{1}\varepsilon +B_{2}\varepsilon^{2}+B_{3}\varepsilon^{3}\right)}=(1+C_{1}\ln\dot{\varepsilon }*)$$

Substituting *A*_1_ and *B*_1_ ~ *B*_3_ gives *C*_1_ = 0.08947.

By introducing the parameter *λ* = *λ*_1_ + *λ*_2_ ln$\dot{\varepsilon }$*, the formula can be transformed into

(11) $$\frac{\sigma }{\left(A_{1}+B_{1}\varepsilon +B_{2}\varepsilon^{2}+B_{3}\varepsilon^{3})(1+C_{1}\ln\dot{\varepsilon }*\right)}=\exp[(\lambda_{1}+\lambda_{2}\ln\dot{\varepsilon }*)(T-T_{r})]$$

Taking logarithms on both sides,

(12) $$\ln[\frac{\sigma }{\left(A_{1}+B_{1}\varepsilon +B_{2}\varepsilon^{2}+B_{3}\varepsilon^{3})(1+C_{1}\ln\dot{\varepsilon }*\right)}]=\lambda (T-T_{r})$$

The value of *λ* is obtained through curve fitting, and further fitting *λ* ~ ln$\dot{\varepsilon }$* gives *λ*_1_ = −0.00716 and *λ*_2_ = 0.0012.

Using the above converted values and applying them to each parameter, the ultimate modified JC constitutive model for the base metal is

(13) $$\begin{array}{l} \sigma =(75.06529+500.4209\varepsilon -2831.127\varepsilon^{2}+6062.14381\varepsilon^{3})(1+0.08947\ln\dot{\varepsilon }*)\\ \exp[(-0.00716+0.0012\ln\dot{\varepsilon }*)(T-T_{r})] \end{array}$$

**Figure 10:**
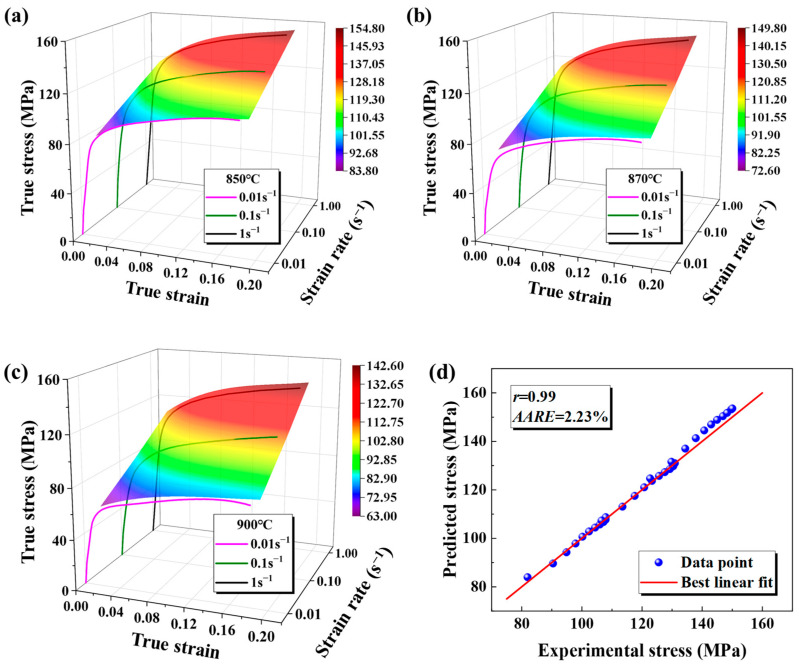
The fitting results and the model’s prediction accuracy for the modified JC constitutive model under different conditions in the base metal are as follows: (**a**) 850 °C; (**b**) 870 °C; (**c**) 900 °C; and (**d**) the modified JC constitutive model’s prediction accuracy.

Figure 10 displays the curve fitting results for the modified JC model on the base metal, evaluated under different temperature and strain rate conditions. The modified JC model matches the flow curve for the base metal better than the original. The correlation factor, *r*, equals 0.990, accompanied by an *AARE* of 2.23%.

Analogously, the modified JC relation for the weld is

(14) $$\begin{array}{l} \sigma =(58.18571+858.7758\varepsilon -6168.2947\varepsilon^{2}+16751.12004\varepsilon^{3})\\ \left(1+0.0436\ln\dot{\varepsilon }*)\exp[(-0.00367-0.000069687\ln\dot{\varepsilon }*)(T-T_{r})\right] \end{array}$$

**Figure 11:**
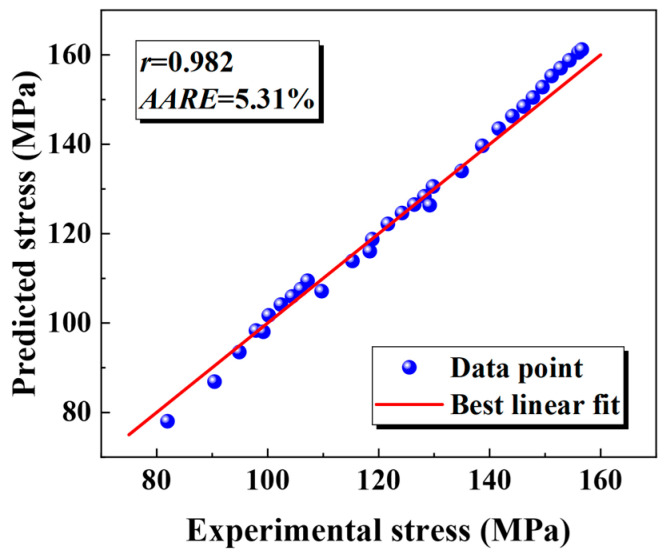
The prediction accuracy of the modified JC constitutive model for the weld.

As illustrated in Figure 11, the modified JC constitutive model demonstrates the strong ability to accurately represent the behavior of the weld. The modified JC model closely fits the data for the weld with *r* = 0.982 and the *AARE* = 5.31%.

## 4. The Application of the Developed Constitutive Model for Complex Tubular Parts

### 4.1. Establishing the Experiment and the Simulation Model

#### 4.1.1. Hot Gas Bulging of Complex-Shaped Tubular Parts

**Figure 12:**
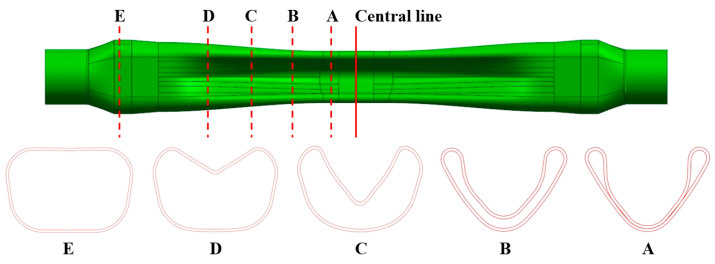
Complex-shaped tubular part and typical cross-section.

The hot gas bulging process for boron steel enables the production of complex-shaped tubular parts, including A-pillars, roof beams, and torsion beams. In order to verify the accuracy of the modified JC constitutive model in predicting the deformation behavior of welded tubes during hot forming and promote its application in production, a tubular part with a long strip and cross-section was designed based on the structural characteristics of automobile torsion beams, as shown in Figure 12. The part contains five typical sections, where the bulging rate of section C is 13%, and there are smaller rounded corners, which potentially indicate a poor fit and a significant reduction in the wall thickness.

**Figure 13:**
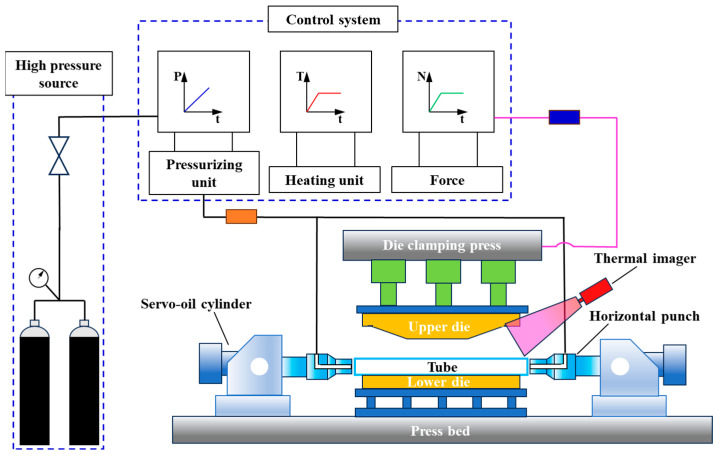
Hot gas bulging experimental setup.

The hot gas bulging experimental setup for the PHS1500 welded tube with a complex shape is shown in Figure 13. This setup mainly includes a press, upper and lower dies, a control system, a heating unit, a temperature measurement unit, and a pressurization unit. The press boasts a clamping capacity of up to 8000 kN, a maximum clamping rate of 180 mm/s, and the ability to transmit 380 kN of axial force through the sealing punch. The maximum bulging pressure of the pressurization unit is 35 MPa, and the control accuracy is 0.1 MPa. In this paper, the heating furnace is used to heat the tube preform, and the thermal imager serves for measuring the temperature during the die closing process. In order to ensure that the tube forming–quenching processes are completed simultaneously, cooling water is passed through the upper and lower dies to ensure the right cooling rate during tube quenching.

The forming process involves several key stages: Initially, the raw tube is heated until it reaches or exceeds the austenitizing temperature. Once properly heated, the tube is swiftly moved into the designated position of the die cavity. The die is then promptly closed and sealed in the axial direction to maintain pressure, and then the tube cavity is pressurized to the set bulging pressure at different pressurization rates. Finally, the die is opened and the component taken out after holding the pressure for 10–15 s.

**Figure 14:**
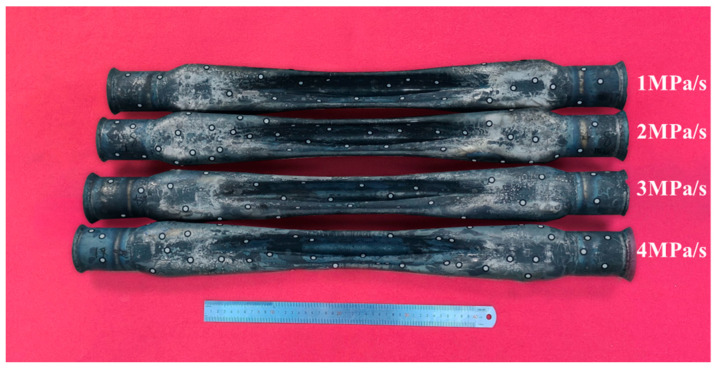
The complex-shaped tubular parts obtained through the experiment under different pressurization rates.

In this experiment, the hot gas bulging law for complex-shaped tubular parts under different pressurization rates is analyzed. The experimental scheme is shown in Table 3, and complex-shaped tubular parts obtained through the experiment under different pressurization rates are shown in Figure 14.

#### 4.1.2. The Finite Element Analysis

**Figure 15:**
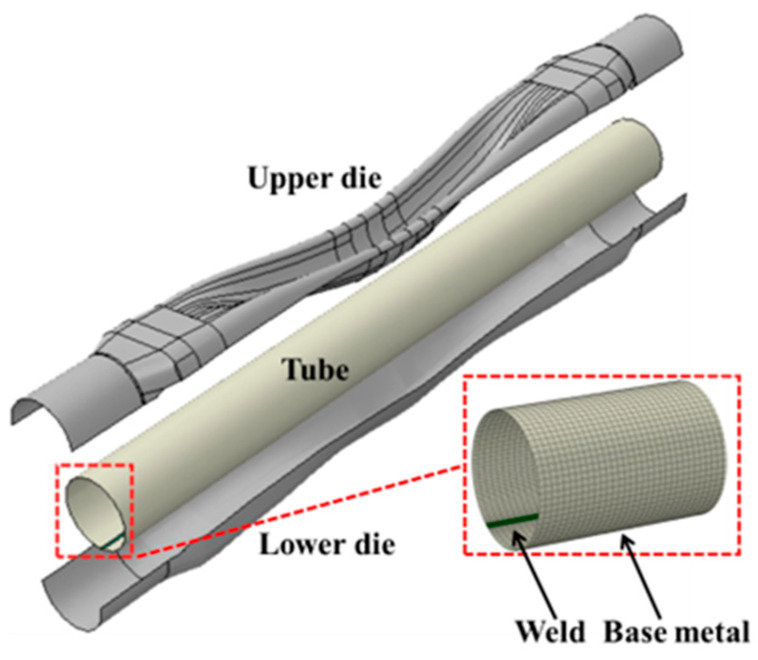
Finite element model establishment and mesh division of hot gas bulging of welded tubes.

To confirm the precision of the proposed modified JC constitutive model for welded tubes, a finite element simulation of hot gas bulging was developed using ABAQUS 6.14 software. The numerical results were then cross-checked against actual experimental data to assess the reliability of the model. The finite element model mainly includes three parts: the upper die, the lower die, and the initial welded tube. The welded tube has a 1.4 mm wall thickness. The upper and lower dies, along with the welded tube, are modeled using shell elements and designated as deformable. Within the interaction module, both the upper and lower dies are designated as rigid entities. The simulation model adopts the thermal–mechanical coupling module in Abaqus and is solved using an explicit solver. The temperature rise due to plastic deformation is calculated using adiabatic approximation. The modified JC constitutive model is adopted as the material model for the base metal and the weld, and its plastic parameters are calculated using the modified JC constitutive equation. The thermophysical parameters of the base metal, the weld, and the die are shown in Table 4, Table 5 and Table 6 [32]. Furthermore, the dies, along with the tube, employ an S4RT grid type. To be specific, the dies have a grid size of 2 mm, and the base metal area of the welded tube also uses a 2 mm grid, while the weld area utilizes a much finer 0.1 mm grid. At the same time, the weld and its adjacent base metal area are meshed, as shown in Figure 15.

**Figure 16:**
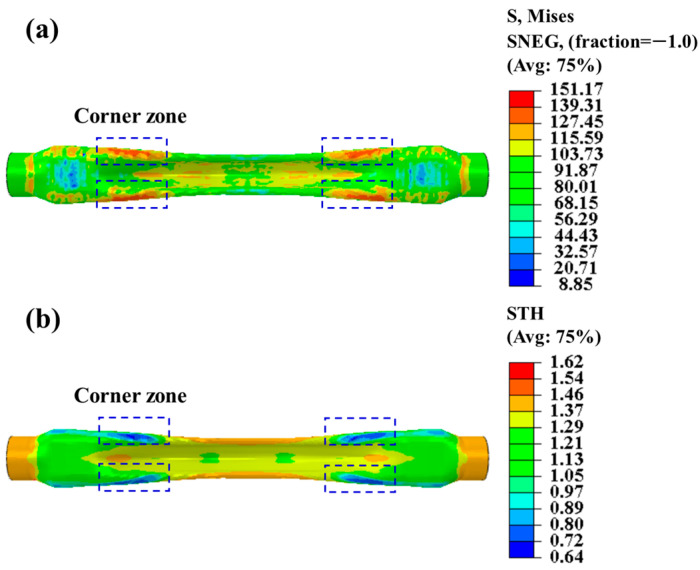
Simulation analysis results at forming temperature of 870 °C, pressurization rate of 3 MPa/s, and bulging pressure of 15 MPa: (**a**) Mises stress; (**b**) wall thickness distribution.

Hot gas bulging formation in boron steel requires the judicious alignment of the forming temperature, the rate of the pressure increase, and the bulging force. For example, when the pressurization rate is high, local stress concentrations may lead to problems such as fractures. Figure 16 shows the distribution of the stress and the wall thickness under the conditions of a forming temperature of 870 °C, a bulging pressure of 15 MPa, and a pressurization rate of 4 MPa/s. Significant stress concentration occurs in the fillet region of the C-section within the complex-shaped tube tubular parts, and the maximum Mises stress is about 140 MPa. At the same time, the wall thickness of the fillet area is seriously thinned, and the minimum wall thickness is thinned to about 0.7 mm. According to the analysis of the stress distribution and the wall thickness distribution, the fillet area of complex-shaped tubular parts is the key area of concern in process parameter optimization.

### 4.2. Prediction of the Wall Thickness Distribution

**Figure 17:**
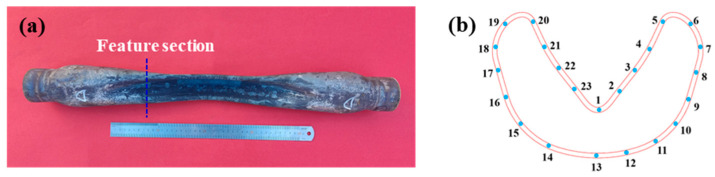
The cutting position of the experimental part and the measurement point of the section wall thickness: (**a**) the cutting position of the C-section; (**b**) C-section wall thickness measurement point.

In the hot gas bulging process for boron steel, measuring the wall thickness of a cross-section of the formed part is the main basis for judging whether this part qualifies or not. When the reduction in the wall thickness exceeds the tolerance threshold, this may lead to premature failure of the part during service. Additionally, variations in the cross-sectional wall thickness of the part reflect the flow uniformity and the stability of the forming process. The local thinning or thickening phenomenon can provide a basis for rational matching of the key process parameters (forming temperature, pressurization rate, and bulging pressure). Hence, with an 870 °C forming temperature, a 15 MPa bulge pressure, and a 4 MPa/s pressurization rate, the C-section wall thickness of the tube was gauged post-formation. A C-section of the experimental part under the condition of the process parameters is cut, and 23 cross-section measurement points are selected to quantitatively characterize the wall thickness reduction, as shown in Figure 17.

**Figure 18:**
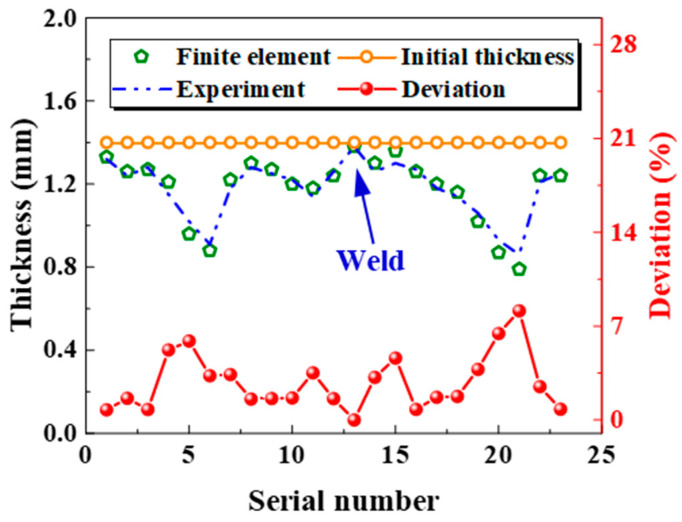
A comparison of the wall thickness between the experimental test and the simulation analysis in the C-section.

Figure 18 compares the experimental and simulated tube wall thickness measurements. It is found that there is a significant thinning phenomenon at the No.5 and No.21 measurement points of the V-shaped side wall in the fillet area, and the No.13 measurement point experiences almost no thinning in the weld area. The cross-section exhibits non-uniform thinning at each measurement point, which is related to the fact that load transfer of the material does not occur in the adjacent area due to the low yield strength and high friction force during hot forming; that is, the material will be thinned due to tension and thickened due to compression. The weld zone resists thinning deformation because of its high strength. By comparing the simulated and experimental wall thickness reduction data, it is found that the two are in good agreement, and the maximum error is less than 8%. The finding demonstrates that modifying the JC constitutive model for hot gas bulging simulations effectively captures the deformation behavior of welded tubes. This shows that the simulation analysis of hot gas bulging through modifying the JC model can accurately simulate the deformation behavior characteristics of welded tubes, offering theoretical support for quality control in hot gas bulging of welded tubes. This approach offers a theoretical framework for ensuring the quality of hot gas bulging of welded tubes.

## 5. Conclusions

(1) Under thermal conditions ranging from 850 °C to 900 °C and strain rates spanning 0.01 s^−1^ to 1 s^−1^, the base metal and the weld of a PHS1500 non-uniform welded tube demonstrate a progressively higher yield and tensile strength as the strain rates escalate. The yield strength of the weld surpasses that of the base metal by 12.8%, while the tensile strength increases by 3.9%. Furthermore, the tensile strength in the base metal and the weld zone exhibits an approximate 11% higher susceptibility to the strain rate compared to that of the yield strength.
(2) Utilizing the elevated-temperature flow curve for the base metal and the weld of a PHS1500 welded tube, the JC material model was formulated and modified. The resulting modified model provides a much tighter fit to the data, showing greater accuracy than the original. The new model demonstrates a marked improvement over the traditional one. Specifically, the correlation coefficients for both the base metal and the weld jump from 0.975 and 0.980 to 0.990 and 0.982, respectively. Furthermore, the average relative error is slashed from 10.23% and 8.75% down to a mere 2.23% and 5.31%, showcasing a significant boost in accuracy.
(3) The modified JC constitutive model is applied to a finite element analysis of hot gas bulging of a complex-shaped tubular part. The maximum deviation between the experimental test of the reduction in wall thickness and the simulation analysis of the cross-section of the complex-shaped tube is less than 8%, which proves the accuracy of the modified Johnson–Cook constitutive model. The modified constitutive model is suitable for analyzing the deformation behavior of other hot stamping steels under thermal loading conditions and is not limited to the hot air expansion process.

## Figures and Tables

**Table 1:** Chemical composition of PHS1500 boron steel sheet (wt%).

C	Si	Mn	P	S	Al	B
0.23	0.15	1.36	0.012	0.002	0.05	0.0018

**Table 2:** The parameters of the JC constitutive model of the parent material.

*A*	*B*	*C*	*n*	*m*
75	117.085	0.09153	0.669	0.963

**Table 3:** Experimental scheme of hot gas bulging of tubes.

Forming Temperature (°C)	Bulging Pressure (MPa)	Rate of Pressurization (MPa/s)
870	15	1, 2, 3, 4

**Table 4:** Thermal conductivity of base metal and weld.

**Temperature (** **°C** **)**	25	150	250	400	600	900
**Thermal conductivity (W/m/** **°C** **)**	31	32	36	41	44	45

**Table 5:** Specific heat capacity of base metal and weld.

**Temperature (°C)**	25	150	250	400	600	900
**Specific heat capacity (W/m/°C)**	469	470	473	474	776	1023

**Table 6:** Thermal conductivity of dies.

**Temperature (°C)**	25	150	250	400	600	900
**Thermal conductivity (W/m/°C)**	20	23	24	26	28	31

## Data Availability

The original contributions presented in this study are included in the article. Further inquiries can be directed to the corresponding author.
